# A Study of the Records of One Hundred and Fifty-Five Cases of Operation for Appendicitis

**Published:** 1905-12

**Authors:** Charles A. Morton

**Affiliations:** Professor of Surgery in University College, Bristol; Surgeon to the Bristol General Hospital, and the Bristol Royal Hospital for Sick Children and Women


### A STUDY OF THE RECORDS OF ONE HUNDRED AND FIFTY-FIVE CASES OF OPERATION FOR APPENDICITIS.

BY

CHARLES A. MORTON, F.R.C.S.,

*Professor of Surgery in University College, Bristol; Surgeon to the Bristol General Hospital, and the Bristol Royal Hospital for Sick Children and Women.*

*(Continued from p. 230.)*

---

PERHAPS the most interesting part of this paper will be a confession of my mistakes in the diagnosis of appendicitis. I have once operated for acute appendicitis and failed to find it. In another case I found a pyosalpinx instead, which I removed; I need hardly say this was not to be regretted. In an elderly woman a hard lump seemed more likely to be a malignant growth of the colon, but turned out to be chronic suppurative appendicitis. I have already alluded to a case of large extra-peritoneal abscess, the nature of which was very puzzling. It was sent to me as a sarcoma in the loin. I could not make up my mind for some time between an inflammatory swelling and a new growth. I have operated on at least one case of suppurative appendicitis previously under treatment by another medical man for typhoid fever, but I have made the mistake myself of operating on a case of typhoid fever as one of acute appendicitis. Both this case, and the one in which I found no lesion at all, are so unusual and instructive that I will give an abstract of them.

Mr. Barker [1] records several cases of very instructive mistakes in the diagnosis of appendicitis; and in *St. Bartholomew's Hospital Reports*, 1899, p. 228 are the records of two cases in which operation was undertaken for acute appendicitis and nothing abnormal found. In one case "for severe symptoms."

I may also say that in one or two of my cases of operation for recurrent attacks I have failed to satisfy myself that there was distinct evidence that the attacks had been appendicitis.[2]

[1] *Brit. M. J.*, 1903, i. 477.

[2] See reference to Mr. Bowlby's paper after record of case 1.

CASE 1. *Symptoms of acute appendicitis; no lesion found.* I was asked to see a young man, aged 20, in consultation as a case of appendicitis. On September 10th he had taken some senna, and had abdominal pain and vomiting. There was of course nothing remarkable about that; but on the 12th, without taking any medicine, severe pain in the abdomen came on, and he was "doubled up" with it. He had to leave work, and go home and go to bed. It was better on the 14th, but became severe again at 8 a.m. on the 15th, and was so when his doctor saw him at midday. When I saw him, at 1.30, his temperature was 101°. He looked ill, with dry tongue, pulse 100. There was no distension of the abdomen, but it was tender in the lower part, and especially on the right side, there was no swelling. Per rectum, there was no tenderness or swelling. Late in the afternoon his temperature rose to 103°, and I operated (in the hospital) in the evening. I thought there was an appendicitis somewhere near the pelvic brim, but failed to find any evidence of disease on the outside of the appendix. I did not remove it. Perhaps if I had I might have found ulceration within, as in the case I have referred to in connection with portal pyæmia, in which the temperature was 108°, and yet there was not much more than ulceration within the appendix. This case could not have been typhoid fever, as he got quite well directly after the operation.

In a lecture published in the *Lancet* for July 9th, 1904, Mr. Bowlby refers to two cases under his own care, in which after several attacks, very little wrong was found with the removed appendices, certainly no sign of past inflammation, and yet in one of the attacks the temperature rose to 102.6°. He thinks that the symptoms in these cases are due to temporary distention of the appendix; but if so, why the temperature? In my case at the time of the attack there was no distention. Perhaps in time we shall understand the nature of these cases.

Case 2. *A case of typhoid fever simulating appendicitis.* A young man, aged 22, was sent to the hospital on November 7th last as a case of appendicitis. A week before he began to suffer from headache, but had no abdominal pain until November 3rd, and then when at work such severe pain came on in the lower abdomen as to "double him up." This pain continued, and became "unbearable," and kept him awake at night, but did not lay him up, and he walked up Park Street on the 5th. There had been no vomiting at any time, but diarrhœa had been present for a few days before admission. No epistaxis had occurred. I saw him at 8 p.m. the evening of admission. When asked where the pain started and also where it was then he pointed to McBurney's spot, but said he had had a little in his epigastrum. Temperature 103°, pulse 100. There was no distention or general tenderness of the abdomen, but a tender, defined lump in the appendix region was present. I carefully considered the possibility of the case being one of typhoid fever, as there had been early headache and marked diarrhœa, but I thought the severity of the pain, and the presence of a definite lump (not merely distended coils) in the appendix region, so greatly against such a diagnosis, that I decided to operate at once. On opening the abdomen I found a considerable amount of clear serous fluid, and then I noticed two glands the size of small marbles loosely attached to the cæcum, and then a hard mass just on the inner side of the cæcum, and on bringing this out through the wound it was found to be the last two inches of the ileum very greatly swollen, and dark in colour. It felt almost as if the lumen had been obliterated from infiltration of its wall, and from it a mass could be felt projecting into the cæcum just like the apex of an intussusception, but there was no entering bowel, and it was evidently the enormously swollen mucous membrane of the ileo-cæcal valve. Two inches above the junction the ileum began to decrease in size, but was somewhat swollen for many more inches, and I could feel greatly thickened patches on the surface opposite to the attachment of the mesentery. There was a gland as large as a hen's egg under the peritoneum close to the cæcum. This and the swollen lower end of the ileum formed the swelling felt through the abdominal wall. I could make no certain diagnosis. I had never heard of such a condition of things in typhoid fever, but the patches of thickening along the ileum certainly suggested ulcers. The appendix was not affected. He was none the worse for the operation (pulse was 104 at end), and he remained in the same condition until the third day after, the pain persisting. On the third day the diarrhœa was troublesome, small black clots were passed with the motions, frequent vomiting set in, and he became jaundiced. No Widal re-action could be obtained. The next day the jaundice was very marked; he became delirious, and died on the 12th. There was no enlargement of the spleen or spots at any time. The temperature kept between 102° and 103° after admission, and ran up to 105° before death. Up to the time of his death no definite diagnosis could be made, though we took the precautions usual with typhoid cases. Such marked jaundice is most unusual in typhoid fever. *Post-mortem*. We found the mucous membrane of the last inch or two of the ileum at the valve was almost entirely destroyed by ulceration, and there were numerous ulcers in the lower ileum. No pathological change was found in the liver. The spleen was very slightly enlarged. No serous fluid had recollected in the abdomen. The glands had considerably decreased in size, and the swelling of the lower ileum was much less than during life. Such a condition as I found at the operation is seldom seen on the *post-mortem* table. A considerable quantity of serous effusion is, so far as I know, not known to occur, and was not again present in the case a few days later when the *post-mortem* was made. So much swelling of the lower ileum as I found is again never found *post-mortem*, because the inflammatory swelling has largely subsided after death. But inasmuch as a definite lump was felt through the abdominal wall in this case, and is almost unknown in typhoid fever, the amount of swelling of the lower ileum and glands must have been most unusual. The cause of the jaundice was not explained. The case is a very instructive one as an example of typhoid fever with severe pain of sudden onset, associated with a definite swelling in the appendix region.

With a larger experience of the surgical treatment of appendicitis, I have not altered the opinion I expressed in my former paper, that the safest course to adopt is early operation, and that this should be done in all severe cases at once, because we cannot tell the extent of the mischief or the danger by any bedside signs or symptoms. I care very little as to the actual condition of the appendix, whether it is perforated or gangrenous, or only inflamed and allowing bacterial infection around; the real point of importance is the condition of surrounding infection. I have several times found a gangrenous appendix, shut off in an abscess sac, and on the other hand a minute perforation associated with general infection. No one who adopts the view that all severe cases should be at once operated on—and it is one the adoption of which is becoming quite frequent amongst surgeons now—denies that many cases recover without operation, and therefore in some cases an unnecessary operation will be done. I say in "some cases," because of the many who would recover without operation, subsequent operation would be required in a considerable number for relapse. We freely admit, I say, that this is so, and it is right to explain to patients that we cannot tell whether theirs is a condition from which they will recover without serious risk, or one which if not operated on early may, in spite of late operation, be beyond the control of surgical interference. There are many, of course, who say they are perfectly well able to distinguish the one kind of case from the other at every stage in its progress, but, alas! every now and then they call in the surgeon, or if surgeons, they operate, too late, when the opportunity has gone. I do not say that every severe case of appendicitis should be operated on by inexperienced hands, and under unfavourable conditions as to light, assistants, and nursing, but certainly in conditions favourable to operation it is the safest course. My two fatal cases of diffuse peritonitis, several of my cases of general peritonitis, and all the cases complicated by intestinal obstruction, would almost certainly have recovered if they had been operated on as soon as the diagnosis of appendicitis could have been made. In the fourth case of intestinal obstruction, this would, of course, have been in the previous attack of appendicitis, which caused the adhesions. It is not enough to point to dozens of cases which have recovered from appendicitis without operation; as I have already said, no one denies their existence, though many of them have eventually to be operated on for recurrence. We must recognise the fact that every now and again a patient dies who could have been saved by an early operation, involving almost no risk in experienced hands.

I have seen a few cases of appendicitis in which I have not operated because the condition has been improving, or the patient has declined operation, and they have recovered. Those who see many cases of appendicitis recover without operation must think not only of these, but of the cases of general peritonitis handed over to the surgeon too late: of the cases of large foul abscesses proving fatal in spite of operation, from septicæmia—all cases which would almost certainly have recovered if the appendix had been removed as soon as the diagnosis of appendicitis had been made.

In my previous paper I stated that if the case was improving I should not operate, but that I did not feel quite happy about leaving such cases alone. Further experience has brought additional illustration of general peritonitis supervening when the patient seemed getting convalescent, as well as when the course of the disease seemed so favourable that no operation was considered needful; and other surgeons have published similar experiences of the fallacious signs of improvement, followed by general infection of the peritoneum. I have found a gangrenous patch in an appendix when the pain and tenderness had almost gone, and the case would not have been operated on if portal pyæmia had not supervened; and in another case, in which the whole appendix was gangrenous and full of stinking pus, the pain of the onset had so much subsided that the girl slept the whole night preceding the day of operation, but, without recurrence of any severe pain, there was a rigor and high temperature. As Murphy of Chicago has pointed out, with the onset of complete gangrene the pain disappears. Again and again I have found pus after the subsidence of pain except on movement, and I think even after complete subsidence in some cases. I have operated on account of persisting pyrexia or recurring pyrexia in these cases. All tenderness as well as pain may disappear from an appendix abscess, and then the abscess may rapidly enlarge, with re-appearance of pain, or may give way and infect the general peritoneal cavity. Whilst writing this paper I have heard of a very striking case, which confirms what I have written. A case of appendicitis in the second attack seemed progressing so satisfactorily towards convalescence that an operation to remove the appendix to prevent further attacks was only undertaken at this early period as a matter of convenience. The patient had been free from pain, and the temperature normal for three days, and yet a gangrenous appendix was found in an abscess sac. There are no distinctive signs of the presence of pus at an early stage of appendicitis, and it is useless to endeavour to decide if pus is present or not. If the signs and symptoms of appendicitis are marked the appendix should be removed. Without any pus outside, and without protective adhesions, I have seen the appendix tensely distended with stinking pus, and yet the pain, which had been very severe, was for some hours before operation present only when the patient moved about in bed.

If operation is undertaken to prevent recurrence—and now a large number of surgeons advise operation for this purpose after even one attack—it is usually recommended that it should be put off until convalescence from the acute attack is complete. This means that the patient has to lay up again some weeks for the operation. I myself think that unless there is marked general distension of coils, which undoubtedly adds to the difficulty of removal of the appendix, there is no reason why the appendix should not be removed as well in, as after, the attack, and the risk of that attack removed, as well as further attacks prevented. If we find pus it may not be well to remove the appendix, but then we should feel thankful we had operated, because we could evacuate the pus. One objection I have heard urged against operation during the attack to prevent recurrence was that the inflamed bowel is likely to tear. If the bowel around the appendix is so much damaged as to be likely to tear with gentle manipulation, then it seems to me evident—having regard to the immediate safety of the patient—that the sooner that appendix is removed the better. Certainly the removal of the appendix during the attack may necessitate the use of a drainage tube, but this need only be quite small, and the opening for it not large enough to really weaken the scar. Moreover, I use a very fine drainage tube in all cases operated on in the quiescent period.

I cannot conclude this paper—though I fear I have already made it too lengthy—without a reference to the rather burning question of the day—as to the removal of the appendix in all cases operated on. The only condition in which surgeons do not remove it is when it lies in the wall of an abscess sac formed by adhesions of intestinal coils, and well shut off from the general peritoneal cavity. Many of us refuse to take the risk of disturbing such adhesions, in order to isolate it and remove it. I have removed it in many cases of localised suppuration in which it has been readily reached, or in which there have been no adhesions, and the pus has been simply kept from diffusion by the presence of surrounding distended coils, or in which adhesions have been so feeble and imperfect as to be of little protective value; but I do not feel inclined to break down well-formed protective adhesions for the purpose. I do not think it gives rise to later trouble sufficiently often to justify the risk.[1] I think I have only had to remove the appendix for recurrent attacks twice after draining an appendix abscess in my 62 cases, but I have had to remove it for persistent suppuration in two cases, and in one case, such removal being refused, the

[1] Mr. Barling (*Brit. M. J.*, 1902, i. 455) records 47 cases of appendix abscess (all traced) in which the appendix was not removed, and of these only one had recurrent attacks.

patient died from persistent suppuration, and in one after an abscess had been drained and the wound healed further mischief in the appendix led to fatal peritonitis. In one case there were recurrent attacks but no operation was performed. I have not been able to follow up my cases yet, and try to discover whether any other patients have had to have the appendix removed, and therefore there may be more cases in which further trouble has occurred than I know of; but these are all the cases I can remember in which such trouble arose, with the exception of a few cases of persistence of a sinus for some time after operation. We must remember that when we open one of these abscess cavities which are well shut off we may see the terminal inch or so of the appendix lying almost free in it, and think it is a case in which we can remove the appendix without seriously destroying the surrounding adhesions; and yet the base of the appendix may be attached to the cæcum, well outside the abscess cavity, and in order to remove the whole appendix, or even all the evidently diseased part, it may be necessary to destroy all the protective adhesions. It may be that I am too conservative in this matter, and that if I write anything more on the question I may advocate always breaking down these protective adhesions, after, of course, most careful packing off with gauze, but at present I do not feel inclined to do so. It is interesting to note that Perrier (quoted by Ballance, *Brit. M. J.*, July 15th, 1905), who removes the appendix in all cases, has not increased his operative mortality by so doing. I referred to the experience of other surgeons with regard to this matter in the *Bristol M.-Chir. J.*, 1902, xx. 158.

I have myself in one case, during removal of the appendix which lay in the wall of an abscess sac, opened up two more abscesses, which I should never have discovered unless I had removed the appendix.

